# Sources, Degradation, Ingestion and Effects of Microplastics on Humans: A Review

**DOI:** 10.3390/toxics11090747

**Published:** 2023-09-01

**Authors:** Yan-Duan Lin, Ping-Hsiu Huang, Yu-Wei Chen, Chang-Wei Hsieh, You-Lin Tain, Bao-Hong Lee, Chih-Yao Hou, Ming-Kuei Shih

**Affiliations:** 1 Department of Seafood Science, College of Hydrosphere, National Kaohsiung University of Science and Technology, Kaohsiung 81157, Taiwan; karta1589483@gmail.com (Y.-D.L.); chihyaohou@gmail.com (C.-Y.H.); 2 School of Food, Jiangsu Food and Pharmaceutical Science College, No.4, Meicheng Road, Higher Education Park, Huai’an 223003, China; hugh0530@gmail.com; 3 Department of Food Science and Biotechnology, National Chung Hsing University, Taichung 40227, Taiwan; naosa720928@gmail.com (Y.-W.C.); welson@nchu.edu.tw (C.-W.H.); 4 Department of Pediatrics, Kaohsiung Chang Gung Memorial Hospital, Kaohsiung 83301, Taiwan; tainyl@hotmail.com; 5 Department of Medical Research, China Medical University Hospital, Taichung 40447, Taiwan; 6 Institute for Translational Research in Biomedicine, Kaohsiung Chang Gung Memorial Hospital, Kaohsiung 83301, Taiwan; 7 College of Medicine, Chang Gung University, Taoyuan 33305, Taiwan; 8 Department of Horticulture, National Chiayi University, Chiayi 60004, Taiwan; bhlee@mail.ncyu.edu.tw; 9 Graduate Institute of Food Culture and Innovation, National Kaohsiung University of Hospitality and Tourism, Kaohsiung 812301, Taiwan

**Keywords:** plastics reduction, microplastics, environmental pollution, hazards, food chain, health

## Abstract

Celluloid, the predecessor to plastic, was synthesized in 1869, and due to technological advancements, plastic products appear to be ubiquitous in daily life. The massive production, rampant usage, and inadequate disposal of plastic products have led to severe environmental pollution. Consequently, reducing the employment of plastic has emerged as a pressing concern for governments globally. This review explores microplastics, including their origins, absorption, and harmful effects on the environment and humans. Several methods exist for breaking down plastics, including thermal, mechanical, light, catalytic, and biological processes. Despite these methods, microplastics (MPs, between 1 and 5 mm in size) continue to be produced during degradation. Acknowledging the significant threat that MPs pose to the environment and human health is imperative. This form of pollution is pervasive in the air and food and infiltrates our bodies through ingestion, inhalation, or skin contact. It is essential to assess the potential hazards that MPs can introduce. There is evidence suggesting that MPs may have negative impacts on different areas of human health. These include the respiratory, gastrointestinal, immune, nervous, and reproductive systems, the liver and organs, the skin, and even the placenta and placental barrier. It is encouraging to see that most of the countries have taken steps to regulate plastic particles. These measures aim to reduce plastic usage, which is essential today. At the same time, this review summarizes the degradation mechanism of plastics, their impact on human health, and plastic reduction policies worldwide. It provides valuable information for future research on MPs and regulatory development.

## 1. Introduction

### 1.1. History of Plastics

Plastics that are physically and chemically stable are often considered low-cost materials. They balance mechanical and electrical properties, weight, cost, flexibility, and adapted production to final uses. Since their discovery, their procedures and categories have consistently improved, with satisfactory commercial value for economic considerations. Remarkable contributions have been made in various industries, such as food packaging, building, electronics, aerospace, and medicine [1,2,3]. However, natural polymers have been used for centuries, with the Mayans recognizing utilizing natural rubber in containers and daily necessities since the 15th century (Table 1) [4]. It was not until the middle of the 19th century (1839) that Charles Goodyear discovered the vulcanization of rubber and made critical advances that transformed natural rubber into an elastic, malleable and helpful engineering material [5]. In 1869, John Wesley Hyatt succeeded in commercializing a semi-synthetic plastic known as celluloid, which solved the issue of over-harvesting elephants for ivory [6]. However, the celluloid’s high-temperature intolerance and flammability have reduced its usage, making it essential to avoid storage in areas prone to heat to avoid combustion [7,8]. In 1907, Leo Baekeland produced a completely synthetic phenolic resin made from phenol and formaldehyde, which marked the beginning of the application of synthetic polymers in human history [9]. The same year, Leo Hendrik Baekeland improved the phenolic resin process and produced a synthetic plastic called Bakelite, which can decompose slowly in the natural environment [10]. Following this, in 1920, Hermann Staudinger proposed the concept of a covalent macro-molecule: “It is a macromolecular compound in which the chemical geometries of the same monomers are assembled by chemical reactions and linked by chemical bonds,” such as natural and synthetic polymers [11]. In 1926, Waldo Semon synthesized polyvinyl chloride (PVC), with commercial production initiated in the following year, and it was the first plastic to be produced [12,13]. In 1930, Eduard Simon derived polystyrene (PS) from natural resin [14], and in 1933, Eric Fawcett and Reginald Gibson synthesized polyethylene (PE) for one of the first times under high pressure in an ethylene trial [15,16]. Notably, following the outbreak of World War II, the scarcity of the natural polymer supply pushed the exploration of polymer development and accelerated commercial production, together with quality improvement (modifying the chemical structure) and economic access [9,15,17]. In 1954, polypropylene (PP) was first discovered by Karl Rehn and Giulio Natta in the polymerization of crystalline structural regular polymers, which was followed several years later by mass production in Italy [18,19]. In 1967, Nathaniel Wyeth, who worked for DuPont, developed polyethylene terephthalate (PET); the PET bottle has been used for packaging beverages until now, and it was patented for DuPONT DeNEMOURS and Co. in 1973 [20,21]. The materials that are predominantly utilized in the production of plastic films, bags, food packaging, containers, and fishing equipment are PE, PP, and PS [22]. Despite the encouraging potential of natural rubber and polymers, rampant exploitation of rubber and petroleum products has created a double whammy: petroleum resource depletion and the pitfalls of plastics in the environment while affecting the food chain [5,17].

### 1.2. Development of Plastic Particle

Since 1970, scientists have discovered that the oceans contain plastic debris (including shattered and disintegrated debris), where the synthetic polymers (such as macro-plastics (large fragments ranging in diameter from centimeters to several dozen meters) and MPs (1–5 mm plastics and nano plastics (NMPs) < 1 mm)) that pose emerging issues for the environment in terms of pollution, animal ingestion (accumulation of toxins), and being bound by plastics (ropes and bags), thus causing drowning, suffocation, or strangulation [22,23,25,28,29,30,31,32,33,34,35,36,37]. This is also known as a biological invasion due to its known biological effects, as plastics can act as a carrier for the growth of hydrates, diatoms, and bacteria after being transported and drifting [28]. Marine biota face a severe threat from persistent pollutants that are constantly present and are capable of migrating, accumulating, and altering habitats [28,29]. Unfortunately, the concentrations of these MPs appear to have significantly increased on the marine surface over the last few decades and until the 2016 United Nations Environment Assembly (UNEA-2) in Nairobi, Kenya, where MPs were ranked as the most significant scientific issue in environmental and ecological research [24,25,26], resulting in an initiative to encourage countries to consider a ban on single-use plastics, to enhance education and awareness of marine litter by including marine-litter-related elements into educational curricula at all levels, and to achieve sustainable development goals [24]. Subsequently, in 2022, UNEA-5.2 also negotiated a legally binding treaty aimed at the effectiveness of plastic reduction, reuse, and recycling actions, the trade-offs among them, and the expansion of producer responsibility with the expectations of ending plastic pollution by 2024 [38] and solving the issue through a multidisciplinary approach [22].

In addition, the synthetic polymers reported in 104 records included PE, polyamide (PA, including nylon), PP, PS, and PET [22], which can be degraded to form large-sized plastics (>25 mm), medium-sized plastics (5–25 mm), MPs (1 µm–5 mm), and nano plastics (inferior to 1 µm), in descending order of diameter (Table 2) [39,40,41]. Plastics can be categorized into primary and secondary MPs according to their degradation patterns [22,42,43]. The former is purposefully industrially manufactured as plastic fibers or particles in the micron size range and is used to manufacture products such as facial cleansers, cosmetics, or airborne media. Due to the purpose for which these plastic particles have been used, they have mainly been discharged into the environment via wastewater stations [44]. The latter results from plastics of larger plastic sizes that are broken down from various plastic products through degradation (the physical action of sunlight and waves) [45,46]. Conceivably, the inability of plastic particles to completely or gradually disintegrate has resulted in their ubiquitous presence in the marine environment, as described above, and even in mountains, caves, deserts, and canyons on the mainland [45,46].

Negrete Velasco et al. [47] reported a 97% removal of MPs by the treatment process of significant drinking water treatment plants. In contrast, the potential annual intake from drinking water at the average consumer consumption of 1.5 L per day is close to 1 MP (≥63 µm). Still, smaller sizes (<63 µm) cannot be measured, which requires more advanced FTIR and Raman spectroscopy investigations. In addition, the filtration and separation membranes (usually made of plastic materials) also needed investigation.

### 1.3. Plastic Particle Sources

**Figure 1:**
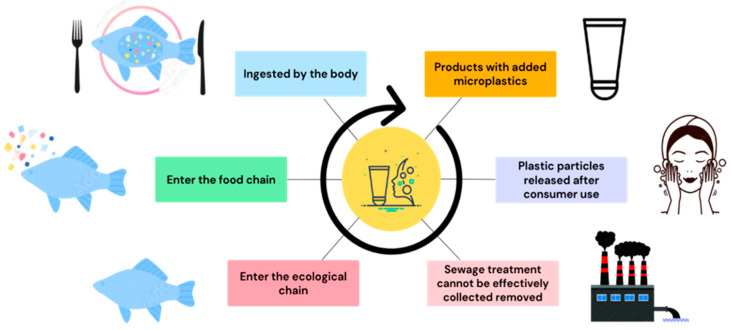
Products with plastic microbeads and the impacts arising from the usage.

The life cycle stages of plastic particles are in the following order: manufacture, transportation, use, recycling, and treatment [48]. They cannot be effectively collected and removed at wastewater stations due to the small particle sizes involved, resulting in those plastic particles being discharged into the waters (Figure 1). In 2017, the International Union for the Conservation of Nature (IUCN) also reported that plastic particles from daily life occur due to human activities, such as the release of fibers from washing clothes, emission of plastic particles from tire wear in transportation, release of plastic particles from furniture that have been rubbed or naturally detached, the release of plastic debris from the processing or transportation of plastics, and the occurrence of plastic beads that are commonly found in skincare products [49,50]. Notably, the World Health Organization (WHO) has also reported that plastic particles travel through different channels, such as the discharge of effluents and washing deposits on the ground (e.g., groundwater rivers, etc.) [51,52,53]. Moreover, wind can carry primary MPs generated through natural degradation or mechanical abrasion into the air and then spread [54,55,56]. Apart from that, Dris et al. [57] reported the co-occurrence of different MPs in the surrounding air, such as dust or synthetic fibers in clothing, indicating that one of the atmosphere’s primary pollutants truly comes from MPs.

### 1.4. Degradation of Plastic Particle

The degradation of plastic materials is due to environmental factors such as mechanical stress, heat intensity, chemical composition, UV radiation, and biodegradation [58,59,60,61]. This process, known as polymer degradation, changes polymer chains’ chemical and physical structures, ultimately decomposing them into smaller debris and molecules [62]. However, traditional plastics degrade exceptionally slowly—and are, therefore, not permanent—but take up to thousands of years, depending on their degradation rate [63].

#### 1.4.1. Physical Degradation

Physical degradation refers to the process in which polymers are fractured or structurally altered from large molecules to small molecules through the breakage of carbon chains due to different biological and environmental factors, e.g., shedding, crushing, and peeling off of plastics [61]. The advantages of physical degradation include ease of operation, shorter time consumption, and energy recovery, while the disadvantages are that it is not easy to degrade polymers completely; thermal degradation might produce toxic gases, which is another issue due to dioxin [64]. Therefore, physical degradation is suitable with other methods, resulting in better performance than a single method [63]. It is essential to understand that the time taken for degradation depends on several environmental factors, which include temperature changes, types of plastic, different strains of microorganisms that aid in degradation, variations in the kinetic of biodegradation, and differences in the amount of energy supplied [65,66,67]. In addition, the physicochemical properties of the polymer material, such as the glass transition temperature, degree of crystallinity, and melting temperature [68,69], also affect the degree of degradation [70]. Temperature changes are utilized to enhance the kinetic energy within the polymer molecules, thus accelerating structural changes or chain breakage and ultimately prompting the polymer to be degraded [71,72,73]. It is worth mentioning that thermal degradation showed favorable performance in processing MPs in wastewater and sludge [74,75]. The degradation efficiency depends on fluctuations in the pyrolysis temperature fluctuations [76], whereas the aging of the plastic during the thermal degradation process may also cause structural defects [77]. Notably, it has been indicated that high ambient temperatures contribute to plastic degradation in topsoil, which includes small plastics (< 5 mm) and MPs [75]. Therefore, the environmental factors favoring the plastic particles during the thermal degradation process require further research so that co-pyrolysis, which refers to the mentioned temperature range with various substances that affect the substances produced afterwards, can be performed [74]. Mechanical degradation, including wind, rain, or friction, primarily utilizes external forces to break the bonds of plastic particles [78,79], while oxygen, temperature, salt concentration in the water, and sediment size may also be influential factors [61,75]. Mekaru [80] reported that micron-sized plastic particles can be degraded to the nanometer scale at room temperature through agitation, which involves friction generated by the collision of water and MPs. In addition, Cesa et al. [81] reported that the proportion of fibers released from synthetic fiber clothing after washing via a washing machine (mechanical degradation) ranged from 0.03% to 0.2% of the mass percentage. Huang et al. [82] reported that after immersing them in water for two weeks, PS cellulose and PE film lost 8.52% and 0.48%, respectively, in weight.

#### 1.4.2. Chemical Degradation

Chemical degradation involves the addition of extra chemicals (e.g., peroxides and carbonyls) during the reaction process to cause chain breakage, oxidation, or cross-linking of the polymer, and this can decrease the polymer’s molecular weight and the physical properties of the polymer material, thus achieving degradation [61]. At the same time, the advantage of means chemical reactions and thus light acts as a physical process. Then, the light can act as a catalysator by furnishing a thermal gradient and/or inducing photochemical reactions. It is based on natural or ultraviolet light, while the disadvantages include its high cost, low degradation efficiency, and the complex preparation of catalysts, which limits its application in practical large-scale production [64]. Therefore, research on chemical degradation methods is focused on developing catalysts that can be easily prepared, with a low cost and high degradation efficiency, while identifying the optimal conditions for chemical degradation, which would be vital for decreasing the environmental impacts of MPs [83]. Photodegradation degradation, which is classified into methods that use light and ultraviolet light, mainly exploits the irradiation of sunlight (wavelengths of 290–400 nm) to stimulate the electronic activity of photosensitizers or photosensitive groups in plastics [78,84,85], thus causing photochemical reactions in the molecular chain and leading to degradation [61]. Its advantages are its eco-friendliness and broad applicability, but its disadvantages are that it is prolonged and easily affected by environmental factors (temperature, humidity, and pH), not complete degradation, notably with different degradation rates for different plastic types. It is worth mentioning that the half-lives of various plastics in the ocean are 38 years for PE, 16 years for PP, 35 years for PS, and 44 years for PET, respectively [86]. In specific situations, the molecular chains of plastic can experience photooxidation (photolysis) due to factors such as temperature, humidity, oxygen, and light [61,85,87,88,89,90]. Specifically, this effect utilizes the free radicals generated in the initiation period after linkage breakage to induce autoxidation, ultimately breaking the plastic particles’ linkages and transforming them into small soluble molecules, which completes the degradation [85,91]. For instance, the light degradation reaction of PET causes ester bond breakage and free radicals to be generated, leading to autoxidation, thus proving that the presence or absence of free radicals facilitates the performance of light degradation [62]. It is essential to consider that light-catalytic substances can accelerate the linkage-breaking process, while the higher the humidity of the environment, the higher the concentration of free radicals, resulting in the same situation [61,63,90].

#### 1.4.3. Biodegradation

Currently, the most attention is paid to biodegradation (circular bioeconomy or biorefineries), which is mainly carried out by using the biochemical reactions of organisms (bacteria and fungi) [92]. The principle is to form a biofilm on the plastic particles as a substrate for growth by utilizing the biofilm that is formed and secreting decomposition enzymes above it for the degradation of the plastic particles, thus either converting or breaking down the chemical structure of the polymers into a simple one through enzymatic or metabolic action [61,93,94,95,96]. Afterwards, microorganisms take in the small molecular compounds and metabolize, combine, or convert them for energy and, eventually, into H_2_O and CO_2_. Similarly to the above, the degradation process is also affected by environmental factors (temperature, humidity, and pH), mainly focused on microbial viability and metabolic efficiency [97]. It is undeniably true that specific secondary metabolites from microorganisms affect changes in pH, thus leading to the breakage of the plastic particles’ bonds, affecting the activity of microorganisms and enzymes, or even leading to bacterial colony changes [98]. However, abundant strains of microorganisms have higher degradation efficiency than a single strain, probably due to the complementary effects of different enzymatic properties. On the contrary, the degradation efficiency decreases when various strains of microorganisms have a competitive relationship with each other [99,100]. Moreover, this requires the consideration of different types of MPs with varying characteristics to effectively select the appropriate microorganism strains for achieving the degradation goal [101]. Auta et al. [102] found that using *Bacillus* and *Rhodococcus* strains significantly decreased PP weight after 40 days of incubation, with reductions of around 4–6%, respectively. A study conducted by Muhonja et al. [103] showed that the weight losses of low-density polyethylene (LDPE) were similar by around 36% after 112 days of incubation of the *Aspergillus oryzae* strain A5 and *Bacillus cereus* strain A5. The weight loss of plastics is an essential indicator of biodegradation efficiency as they are degraded [101,104]. Interestingly, Wang et al. [105] reported that in an investigation of MPs in river shrimp, it was found that microplastics may be partially metabolized through catabolism by the shrimp, leading to an overestimation of their feeding preference for small-sized MPs (containing synthetic fibrous, rayon (RA), PE, and smaller particles (<400 μm)). Despite these advantages of biodegradation, it is crucial to consider the potential hazards of poor degradation for the existing ecosystem, which requires the establishment of a database for analysis and validation before realizing the application scenarios [106]. The pros and cons of the above physical, chemical, and biological degradation methods have been summarized (Table 3) in the hope that valuable information may be provided to aid in the exploration and development of the degradation of plastic particles.

### 1.5. Migration of MPs in the Environment

**Figure 2:**
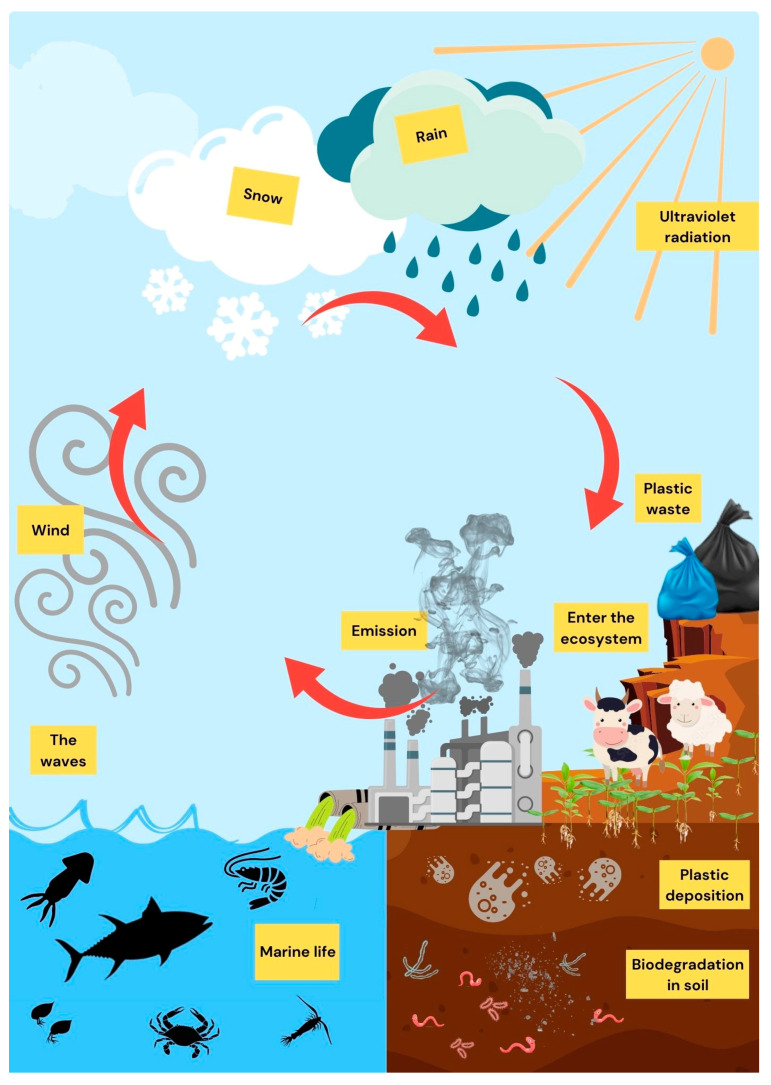
Microplastics migration modes in the environment.

Most people believe that MPs occur on land but end at sea [107] because humans produce plastic debris that is degraded to form MPs, which are subsequently unintentionally carried into the aquatic cycle by the movements of animals, plants, humans, and air, ultimately entering the oceans for deposition [108,109,110]. Notably, a study by Geyer, Jambeck and Law [111] found that only 9–30% of plastic was retrieved, while the rest ended up in the environment. However, not all MPs are eventually deposited in the ocean, as some MPs migrate back to the land from the seas through either the aqueous cycle or the food chain—into animals, plants, and humans (Figure 2). As a significant water pollution source, plastic debris is affected by the environmental factors mentioned in Section 1.3, whereby degradation occurs [112,113,114]. Despite the imposition of large-sized plastic particles in wastewater stations, small-sized plastic particles can enter the oceans despite this interception [51]. Notably, Sharifi and Movahedian Attar [115] reported that drinking water filtration stations in Iran could eliminate 58% and 26% of MPs—of which PP, PE, and PET were the polymers with the highest percentages—in two stages. However, 2.25 × 10^11^ MPs entered the distribution system.

The above treatment has effectively prevented 98% of plastic particles within the 10–500 µm range from entering the environment [116,117]. It is essential to be aware that wastewater from human daily use, agricultural, poultry, livestock farming, aquaculture, and industrial operations may flow into the ocean through groundwater or rivers and that plastic particles may be affected by ocean currents and tides as freshwater enters the intertidal zone along the coasts [33,45,52]. In addition, aquatic biota may have mistakenly ingested plastic particles as food, thus serving as carriers of plastic particles that are then metabolized and transported to different regions of the water or reintegrated into the food chain and returned to the mainland [37,118,119,120]. When plastic particles contact the soil on land, large-sized plastic particles accumulate on the surface, while small-sized plastic particles can easily be infiltrated into the soil layer via the crevices in the soil [121,122]. In addition, Okutan et al. [123] reported that MPs accumulation might be an issue with the actual aquifer instead of its transportation, which required further investigation. Then, plants and soil-growing organisms may ingest the plastic particles [109,110,121,124], followed by their decomposition into MPs, which penetrate deeper into the soil [109,125,126,127] and the food chain through digestion or excretion after ingestion by living organisms (earthworms, fungi and insects) [26,33,128,129]. The soil’s MPs can carry pathogenic bacteria and other pollutants (bisphenols, phthalates, short/medium chain chlorinated paraffin, heavy metals, and persistent organic pollutants) [26,130]. Studies have seriously questioned the assumption that MPs act as chemical carriers, as this phenomenon is negligible compared to their bioaccumulation [37,131,132]. In addition, plastic debris and MPs accumulated on the soil surface and inside the soil may also be subject to degradation and migration due to environmental factors such as pressure, temperature, etc., as mentioned above [37,133].

Regarding air, MPs can also be transported via atmospheric circulation, with different types of plastic particulate debris (fibers, fragments, films, etc.) being detected in the air [22,134,135]; in particular, fibrous plastics have been found at the highest levels [49,136]. In addition, particles can be circulated in the air as aerosol droplets or nano and/or micro fragments [137,138]. The movement of airborne MPs can be influenced by factors such as wind speed, wind direction, and initial concentration; unfortunately, limited information about how these particles are transported in the environment is available [139,140]. However, MPs propagate faster and across longer distances in the air than in soil and water; the average MP concentration in the air is about 1.42 n/m^3^, with around 78% in the upper air layer (80 m above the ground) and 72% is in the lower layer (1.7 m above the ground), which may be related to the density of the polymers [136]. Moreover, airborne polymers may also be deposited on the surface or surface water via deposition [57], with the usual deposition of airborne fibers being at concentrations of about 0.9 n/m^3^; in contrast, with rain, the amount of fiber deposits was elevated five-fold in comparison with that in a rain-free period due to the washing of rainwater [141].

## 2. Risk of Plastic Particles Entering the Human Body via the Food Chain

Since plastic products are convenient, practical, and commercially valuable, the primary source of plastic pollution in foodstuffs commonly encountered today is single-use plastic products (such as bottled, straws, dishware, bags, etc.) or other products with plastic packaging [22]. Therefore, it is necessary to dispose of them properly to avoid environmental pollution rather than just throwing them away. This may even cause plastic particles to be introduced into the food chain through accidental ingestion and absorption by animals and plants [33,110,142,143].

### 2.1. Common Categories of Plastics

Six categories of food plastic products are currently used worldwide for various packaging applications based on their unique characteristics, and these categories include PET, high-density PE (HDPE), PVC, LDPE, PP, and PS [144]. However, most plastics eventually become waste for various reasons, including poor recycling management systems and high treatment costs, making them the predecessors of MPs [144]. According to a report, about 390.7 million plastic products will be manufactured globally in 2021, which will continue to exhibit an increasing trend [145] and triple by 2050 [104]. It is worth mentioning that plastic packaging is mainly used to package perishable food and beverages [146]. Moreover, in recent years, the increase in the use of e-commerce platforms and the boom in food delivery services have led to an increase in the frequency of the use of plastic products, leading to a rise in the production of MPs; these were also associated with the COVID-19 crisis of 2020–2022 [147] and the use of disposable personal protective equipment (face mask, gloves, protective suits, and goggles) [92,148,149].

### 2.2. Common Plastic Particles in Food

Regrettably, numerous MPs have been detected in various foods, including drinking water, seafood, milk, sugar, and salt [3,35,56,143,144,150,151,152,153,154,155,156]. However, plants cultivated on land contaminated with MPs have also been found to absorb MPs, which may enter the food chain following harvest and be ingested into the human body [109].

#### 2.2.1. Water

Water is one of the sources of life for human beings, and the human body must consume water to function. MPs have already contaminated today’s drinking water. Moreover, there is much evidence that mineral water worldwide has been found to contain about 7.4 pieces of MPs per liter [152,157,158]. Simultaneously, this implies that MPs have polluted drinking water; thus, alternative packaging materials for drinking water and bottled beverages should be explored to minimize the contamination with MPs [110,155,159,160].

#### 2.2.2. Milk

Since milk products are widely consumed and frequently used in foods for babies and young children, MPs in food have attracted much attention [161]. According to studies by Diaz-Basantes et al. [162] and Kutralam-Muniasamy et al. [153], the average number of MPs amount in Mexican milk of different brands was found to be about 23 pieces per liter, which could be attributed to the ingestion of MPs in the environment by cows, allowing them to eventually migrate into the milk, assuming there is no adulteration issue. Apart from this, drinking water contaminated with MPs is also one of the crucial sources for the intake of plastic particles [115,163], while a few sources may come from food processing, handling, production, and packaging.

#### 2.2.3. Sugar

Sugar is also a commonly used flavoring in daily life. A study found that commercially available sugar in Bangladesh contained an average of about 344 ± 32 pieces of MPs per kg, with plastic particles less than 300 μm accounting for most of the particles [164]. In contrast, a survey of commercially available sugar in Iran by Makhdoumi et al. [165] showed about 58 ± 21 MPs per kg. Excluding the issues of the sugar manufacturing process and experimental methods, etc., this also shows that the MPs contamination has different levels in each place.

#### 2.2.4. Salt

Salt is a widely used flavoring in daily diets. According to a study by Makhdoumi et al. [165], each kilogram of salt contains an average of 55.2 pieces of MPs, implying that the uncontrolled use of plastics causes serious ocean contamination and potentially harmful effects on humans. In addition, Li et al. [166] reported the occurrence of 13 MPs (with a relative abundance of 256 ± 26 particles/10 g) in crude salt produced through seawater crystallization; 6–112 particles/10 g were found within the final product after a series of process refinements. Another study on the MPs content of sea salt has also reported that products harvested from the Mediterranean Sea generally have lower MPs loads, while consumers of other sea salts can absorb < 3 MPs per year (approximately 4 μg per year) [143]. 

#### 2.2.5. Seafood Food

It is worth mentioning that oysters and mussels have been utilized to be used as filters and serve as biomonitors in water for the removal and monitoring of MPs, whereas they have also been used to provide feedback on the real-time situation (as bioindicators) of MPs or other pollutants in the area [167,168,169]. Bošković et al. [170] reported that mussels harvested from the Adriatic Sea have been found to contain PA, PVC, and PS. Their study highlighted that consuming a single serving of these mussels would lead to an intake of approximately 23 MPs, while it has been estimated that individuals may consume up to 99 MPs per year by including mussels in their regular diet. Interestingly, Cole et al. [171] reported that mussels reduced the number of MPs in the water at an average uptake amount of 40,146 MPs kg^−1^ h^−1^ at an initial concentration of 1000 MPs L^−1^ in a laboratory tank and that mussel feces precipitated at a mean sinking rate of 223–266 m per day, regardless of the MPs content contained. These findings underscore the importance of conducting comprehensive research on the potential health risks of MPs. Hence, mussels are recognized as some of the food products that have been seriously contaminated with MPs [172]; they are a popular seafood food that represents a health hazard for humans who ingest large amounts of MPs through the food chain [155,173].

Interestingly, some commonly consumed fish have been detected with MPs in their digestive tracts but not in the edible parts of the fish because they had been gutted before consumption, thus causing negligible risk of direct exposure [174,175,176,177]. In particular, the sardines, minnows, and brown/brine shrimp, which are typically eaten whole in the food chain, would facilitate the potential transfer and accumulation of MPs within the tops of the food chain [174,178], namely, large fish and filter-fed megafauna [175].

## 3. Impacts of MPs on Human Metabolism

**Figure 3:**
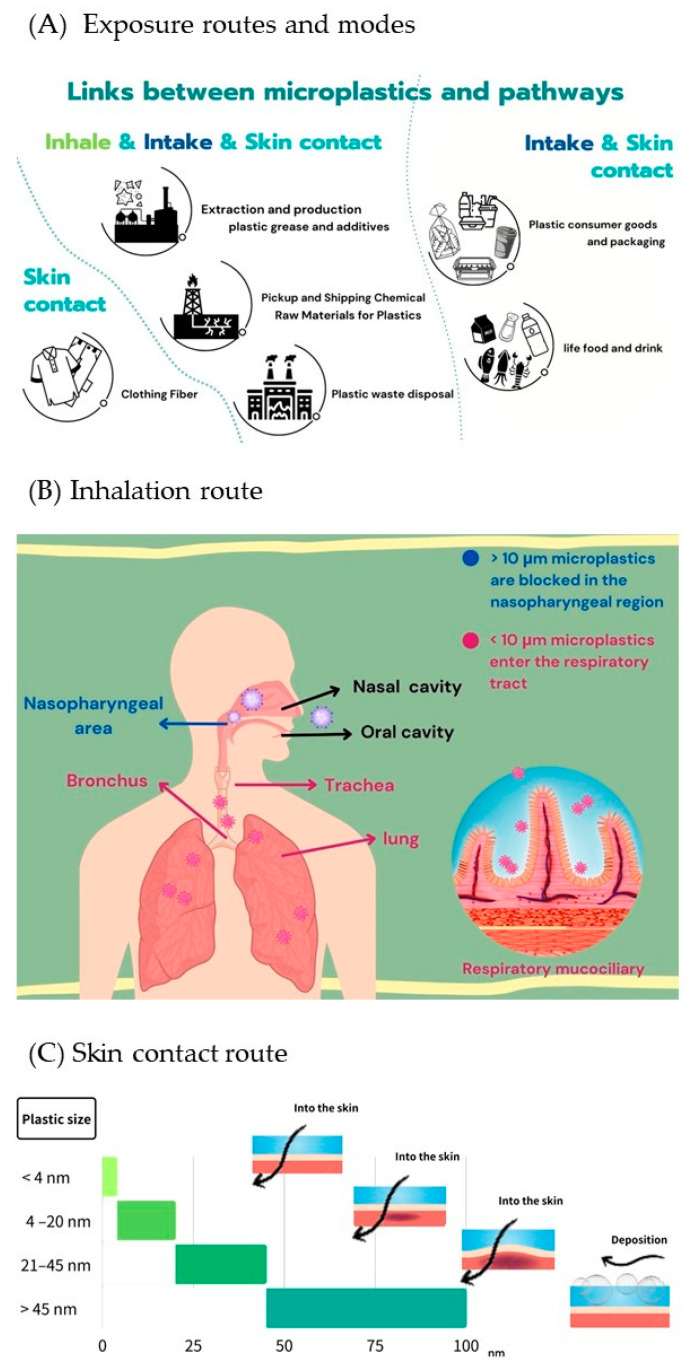
MPs have (**A**) three routes and modes of exposure, including (**B**) the inhalation route and (**C**) the skin contact route.

Due to their small particle size, MPs can enter human tissues and organs in various ways, affecting reproduction, growth, and the immune system [2,156,179]. There are three exposure routes for MPs to enter the human body: ingestion [2,180], inhalation [120,181], and contact with the skin [182] (Figure 3A). The details of the mechanisms involved are described in the following.

### 3.1. Ingestion Route

Ingestion is the predominant means of uptake of MPs by the body, as they eventually enter the body through the food chain [26,156,183], as in the case of the foods mentioned in Section 2. Cox et al. [184] showed that the dose of MPs ingested by humans via air and typically consumed food was estimated to be about 203 to 332 MPs per person daily. Our knowledge of how plastic packaging affects the amount of plastic waste and microplastics in the environment and their potential entry into the human food chain is minimal, and it will require more investigation and analysis for further clarification.

### 3.2. Inhalation Route

Suspended MPs commonly found in the air include PS, PE, PET, pharmaceuticals, personal care products, pesticides, stimulants, and trace metals [57,136,156,185,186]. As previously discussed, MPs refer to tiny particles in the air that can gather in the respiratory system, penetrate the blood-brain barrier (BBB), and potentially harm human health when breathed in or through exposure to contaminated air [140,179,183,187]. Based on surveys, it is estimated that nearly 136,000 tons of plastic particles are released into the air annually and transported through the atmosphere, with a maximum travel distance of 95 km [188]. Notably, indoor environmental pollution is more severe than outdoor pollution [181,183,189,190], whereas an indoor study in Paris showed that the concentration of MPs was about 1–60 n/m^3^ compared to less than 2 n/m^3^ outdoors [141]. Although the human body has mechanisms for shielding against large MPs (>10 μm in diameter) and depositing them in the nasopharyngeal region, they cannot penetrate the trachea and enter the lungs [191]. Moreover, Jenner et al. [192] reported MPs as small as 4 μm in human lung tissue samples (*n* = 13), whereas MPs of >2 mm were also detected. However, MPs below 10 μm are deposited in the thoracic region and contact the gastrointestinal tract via mucosal cilia (Figure 3B).

### 3.3. Skin Contact Route

The National Institute for Occupational Safety and Health regulates exposure limits for workers’ exposure to other airborne particulate matter with a 5 mg/m^3^ (e.g., asbestos or silica dust) [3]. Reports have suggested that MPs can enter our bodies through the air and breathe and settle on the skin [179,193]. It is widely known that human skin is divided into four layers: the stratum corneum, the active dermis, the dermis, and the subcutaneous connective tissue [194]. It is essential to know that skin exposure to MPs can occur through clothing fibers, plastic products, and breathing, while extended exposure to these elements can significantly raise the risk of MPs exposure, which can harm health [119,180,195]. According to studies, contact with plastic particles in the air can lead to exposure through skin residue [179,193].

Moreover, apart from particle size, the properties of microplastics and the skin’s condition play a significant role in their ability to penetrate through it [196]. The top layer of the skin, the stratum corneum, acts as a protective shield against foreign substances and harmful microorganisms. Hence, it is essential to maintain the health of the stratum corneum to ensure adequate protection. According to a report by Larese Filon et al. [197] regarding the movement of nanoparticles on human skin, it was found that particles smaller than 4 nm can easily penetrate through healthy skin. Similarly, particles with a size of between 4 and 20 nm can partially penetrate both healthy and damaged skin. However, 21–45 nm particles can only enter damaged skin. Nanoparticles larger than 45 nm cannot be transferred across the human skin but can be deposited on the skin’s stratum corneum (Figure 3C). It is important to note that mixing skin-penetrating nanoplastics with chemical substances can cause discomfort through nerve activity. However, it is worth mentioning that no studies on the ability of MPs to penetrate human skin have been published. Hence, in Figure 3C, there is a detailed summary of the various exposure routes of MPs that can enter the body, including through ingestion, inhalation, and contact with the skin.

### 3.4. Organs Affected by MPs

**Figure 4:**
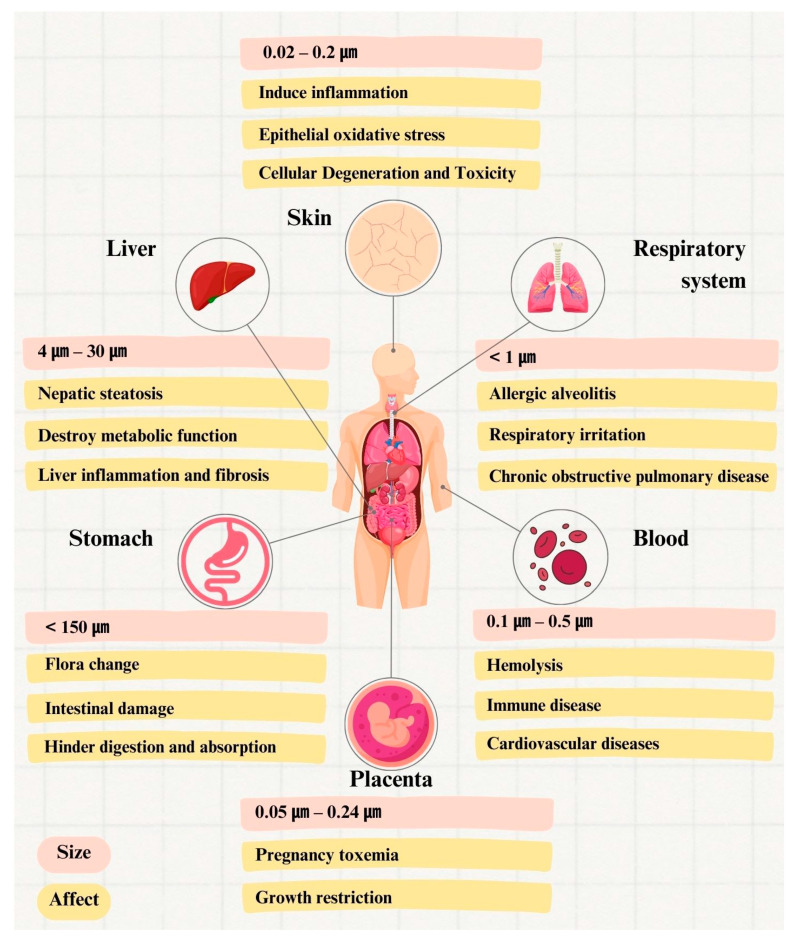
Effect of microplastic particle size on human organs.

MPs have a high surface area, lipophilicity, hydrophobicity, and electrostatic properties, which makes them useful as carriers of certain pollutants, heavy metals, and even toxic substances (pesticide residues), while additives [plasticizer, flame retardant, surfactant bisphenol A (BPA), polycyclic aromatic hydrocarbons (PAHs), and polychlorinated biphenyl (PCB)] are also frequently incorporated into plastics production [3,37,143,150,156,168,173,181,198,199,200,201,202,203]. Notably, Klasios et al. [168] reported the observation of MPs in mussel samples collected from various locations within San Francisco Bay, and the available evidence suggests no correlation between MPs and PAHs. However, MPs can enter the human body through different exposure routes and then spread to various organs and tissues via the respiratory tract, digestive system, or blood circulation. It is crucial to note that the toxins present in the MPs can cause significant damage to the body’s functions (Figure 4) [65,204]. The different particle sizes, shapes, surface charges, concentrations, and exogenous toxins of MPs will have different effects on other organs of the human body, such as oxidative damage, chemical interference, cytotoxicity, inflammation and immune response, DNA damage, changes in the gut microbiome, metabolic disruption and increased risk of inducing immune disorders and cancer [156,173,180,193,198,205,206,207,208,209,210].

#### 3.4.1. Gastrointestinal System

The intestinal tract consists of four parts, from outside to inside: the muscular layer, the mucosal lamina propria, the intestinal epithelial cells, and the plasma membrane layer [189]; these have various roles in digestion, absorption, and metabolism, and they are essentials organ in the human digestive system [211]. It has been shown that humans ingest 0.1–5 g of MPs per week, with a maximum daily dose of 9 mg/kg; most of them enter the body via the gastrointestinal tract, and the particle size of the MPs ingested is less than 0.15 mm [212]. The digestive system takes in macrophages and neutrophils through phagocytosis and vesicle phagocytosis [213,214,215]. Particles with a diameter between 300 and 3000 nm are more likely to pass through Peyer’s patches. In contrast, the villi in the intestinal tract absorb particles with diameters of 50–100 nm, and the surface charge and hydrophilicity of the particles significantly increase their absorption affinity [213,214,216].

Moreover, the multifaceted gut biota, consisting of probiotic, harmful, and harmless microbes with different metabolic and biochemical effects (amino acids, protein synthesis, and high mineral absorption efficiency), has been recognized as a microbial system [217,218]. However, according to Bazeli et al. [219], the daily food ingestion exposure per person is about 583 ng; while small amounts are usually safe, excessive intake of external substances such as MPs or toxins can harm the body’s cells and trigger an immune response [207]. Subsequently, the immune cells will phagocytose the beneficial bacteria, which may cause changes within the gut microbiome composition or the intestinal environment (such as the oxygen content, regular microflora activity, and metabolic activity). It is essential to maintain a healthy balance of microorganisms in the gut for proper digestion and nutrient absorption; at the same time, the impact of MPs on this process should not be underestimated, as it can cause malnutrition and lead to malnourishment, inflammatory bowel disease (IBD) [220], diabetes, and cardiovascular disease [221,222]. Moreover, MPs could quickly enter the mucus layer of the intestines, causing damage to the intestinal barrier or mucosa and resulting in intestinal damage, intestinal perforation, and intestinal blockage [198,223]. Specifically, MPs may enter the circulatory system after crossing the gastrointestinal wall and the intestinal barrier [224,225] or utilize microfold cells in the immune system to enter the bloodstream or lymphatic system through endocytosis and cellular penetration [179,226]. However, this phenomenon may be attributed to the distribution pattern of MPs, whereby the amounts of MPs absorbed are higher than the body’s capacity to absorb MPs, which could potentially be transferred to the circulatory system and distant tissues [227,228,229]. Another possibility might be that the integrity of the barrier cells is compromised, thus enhancing the movement of particles across the intestinal cells [229]. Prata et al. [209] reported that human epithelial cell permeability is elevated during inflammation, and the chance of translocation of MPs also increases. Notably, Thubagere and Reinhard [230] reported that in an in vitro culture of the human intestinal epithelium, treatment with PS nanoparticles induced apoptosis and affected surrounding cells, thus adversely affecting the intestinal membrane.

#### 3.4.2. Respiratory System

The respiratory tract is the pathway through which the human body breathes and exchanges gases [231]. Airborne solids [dust, plastic particles, and most airborne plastic particles (>0.1 mm)] are blocked by mucus fibers [192]. In addition, smaller-diameter plastic particles may still enter the respiratory system via inhalation and the bronchus and may be absorbed through the lung epithelium [232]. However, the defense mechanism of the lungs against pollutants is based on the different particle sizes and densities of MPs [183,233,234]. Specifically, MPs with a particle size of >15 μm are blocked by macrophages and mucosal cilia and deposited in the airways [193]. In the chest region, the diameter of MPs is about 4–10 μm, while the lower respiratory tract requires MPs of <3 μm for passage [235]. MPs’ small size (approximately 1 μm) will allow for easy penetration into and presence in the lungs [193,236,237]. Predictably, the lungs have different MPs uptake and excretion rates and are deposited for different periods [238]. Vianello et al. [239] proved that humans were exposed to MPs in indoor air through respiration in three different apartments by using a respiratory thermal model (*n* = 3), the results of which showed inhalation of MPs in all models, with a maximum size of 11 μm and with an MPs concentration in the range of 1.7–16.2 m^−3^, thus posing a health risk. Simultaneously, excessive concentrations of MPs in the respiratory system may cause adverse reactions in humans, resulting in respiratory damage and leading to respiratory problems of varying severity, including respiratory irritation, lung disease [240], wheezing [241], decreased lung capacity, clinical symptoms similar to allergic alveolitis [181,242] and chronic obstructive pulmonary disease (COPD) [243], and even lead to other diseases [244]. Moreover, workers who inhale asbestos may develop asbestosis and even malignant mesothelioma, apart from the above disease risks [245]. Therefore, long-term exposure to plastic dust, MPs, and nano-plastics in the textile industry can lead to respiratory and occupational diseases and lung cancer [181,193,246,247].

#### 3.4.3. Blood and Immune System

Recent studies have shown that various shapes and sizes of MPs can be found in bodily fluids, and their accumulation may eventually lead to vascular disease [150,248,249]. However, there are still different opinions, as this hypothesis has not been supported by sufficient evidence; for example, there has been a lack of repeated analyses of samples for validation, errors in statistical methods, and accidental contamination in the laboratory processes [250]. In animal studies, it has been reported that small intestinal particles absorbed through the skin and cells migrate to other body tissues through the vasculature; specifically, 1–4% of MPs in the intestine migrate to the bloodstream and are detected in the lymphatic lumen, the translocation of NPs is thought to be significantly less, the most likely sites of accumulation are Peyer’s patches in the small intestine, and the MPs have also been shown to be hemolytic [213,251,252,253]. Moreover, this may also cause platelet aggregation and increase the risk of thrombosis [254,255] and even atherosclerosis, in addition to promoting the development of cardiovascular diseases, such as pulmonary embolism, ischemic stroke, and ischemic heart disease [256,257]. MPs have the potential to impact the immune system negatively. In addition, MPs may induce local and systemic immune responses by activating inflammatory cells and causing subsequent elimination actions. It is crucial to minimize exposure to MPs to maintain optimal immune function and overall health [258].

Moreover, prolonged exposure and the resulting damage can result in chronic inflammation and immune-system-related disorders [such as systemic autoimmune rheumatic disease (SARD) and systemic lupus erythematosus (SLE)] [259,260,261], ultimately increasing cancer risk [209]. However, the possible factors contributing to immune disorders include oxidative stress, translocation of MPs within the body, and immune activation [156,209,260]. It has been indicated that autoimmune diseases develop due to multiple factors. In contrast, those associated with MPs include plastic particle migration, the release of immunomodulators, immune activation, exposure to self-antigens, and the production of autoantibodies [209].

#### 3.4.4. Brain and Nervous System

Although few studies on the harmful effects of MPs on the brain and nervous system have been reported, the evidence from current studies indicates that MPs may indeed be neurotoxic [262] and induce oxidative stress, particularly in the brain and the nervous system [187,263]. Moreover, a cellular model of human T98G and HeLa (cerebral and epithelial) cells revealed that exposure to MPs increased free radicals, thus causing oxidative stress [264]. However, the authors of [265] reported that the neurotoxicity of MPs in aquatic ecosystems was strongly correlated with particle size and exposure time but not with species or MPs’ composition, morphology, and concentration. Simultaneously, Salegio et al. [266] reported that nanoparticles with different properties and sizes can be rapidly distributed into remote regions of the brain via the cerebrospinal fluid. Therefore, even though there is no direct evidence of the actual effects of MPs on the human body, the available evidence mentioned above indicates that this issue requires attention and prevention.

#### 3.4.5. Embryo and Placenta Barrier

**Figure 5:**
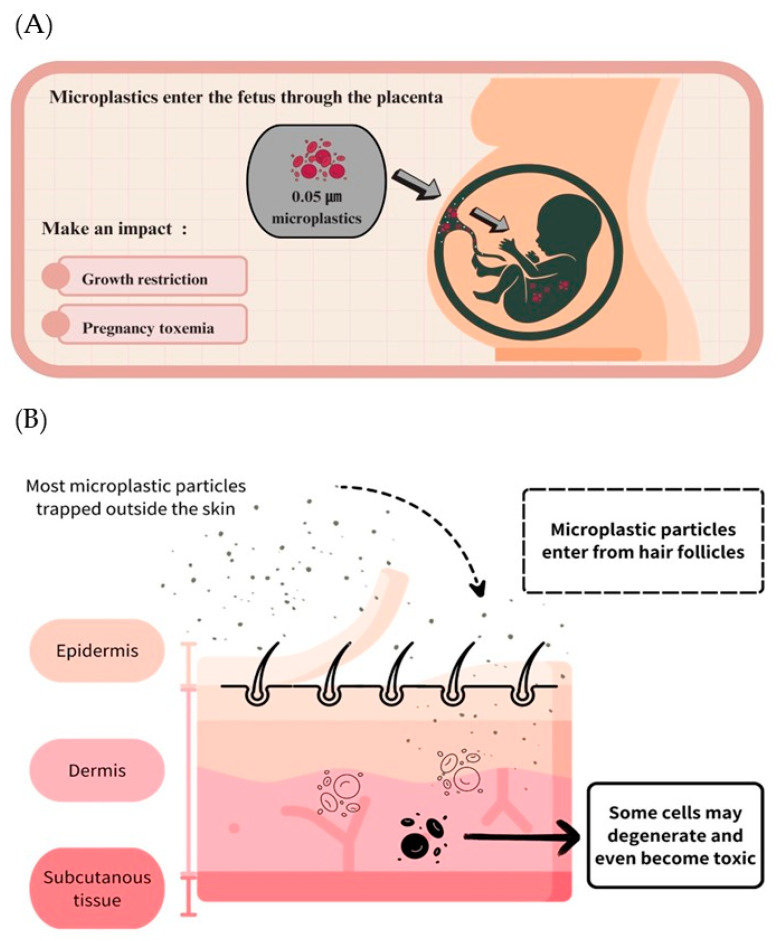
The routes of microplastics into the (**A**) placenta and (**B**) absorption via the skin.

It is widely acknowledged that the placenta plays a vital role in supporting the growth and development of a fetus. Specifically, it is a crucial organ that provides essential nutrients and protects the life developing within a womb [267,268]. Notably, once MPs enter the human body, they are carried to the placenta through internal circulation, and the placenta has a follicular chorionic villus that allows the maternal blood coming into direct contact with the fetus to be absorbed by the fetus [150]. Several studies have shown that MPs have been found in the placenta and even deposited there, whereas the translocation of MPs through diffusion is related to their physicochemical properties (particle size of 50–300 nm and electric charge) [269,270] (Figure 5A). Moreover, Ragusa et al. [150] reported the detection of 5–10 μm MP found in the placenta of pregnant women (*n* = 6, not detected in two) in addition to possible individual differences depending on different physiological conditions and genetic characteristics, as well as in terms of the patients’ various dietary habits and lifestyles. However, as suggested in a study by Braun et al. [271], there may also be a possible presence of early foreign contamination resulting from the samples at sampling time.

Moreover, the most abundant MPs in the placenta, fetal stool, and infant feces were bisphenol A, which accounted for less than 50%, 60%, and 50%, respectively, whereas the predominant MPs in breast milk and infant formulas were polyurethane, which accounted for 53% and 49%, respectively [272]. A recent report by Liu et al. [272] revealed that, after analyzing placentas, infant feces, breast milk, and infant formulas (*n* = 18), over 74% of microplastics were within the 20–50 µm range. At the same time, the result of the questionnaires indicated that MPs intake in pregnant women might result from exposure to detergents or toothpaste and that breastfeeding and using bottles and plastic toys may be a source of contamination for infants. These findings shed light on the potential risks microplastics pose in infant nutrition, and further research is warranted to fully understand their impact. Since there is no definitive evidence of potential adverse health effects of MP on embryos in current studies, it has been hypothesized that there may be effects on growth factor signaling and immunity during pregnancy and even induction of growth restriction and preeclampsia (also known as toxemia of pregnancy) [273].

#### 3.4.6. Reproductive System

There is a shortage of research examining the influence of MPs on the human reproductive system [143]. Nevertheless, considering the results of numerous animal studies, it is plausible to suggest that MPs may have detrimental impacts on human genitalia [274]. Wei et al. [275] reported that female mice appeared more susceptible than males to impaired fertility caused by MPs. In addition, MPs may have adverse effects on offspring, such as weight loss and the risk of metabolic disorders [276]. Despite the current evidence of their harmful effects in vivo, these findings show that it is imperative to comprehensively ascertain the reproductive toxicity, mechanisms, and dose response of MPs [277].

#### 3.4.7. Liver

It has been reported that MPs (4–30 μm) circulate in the body for translocation to the liver [278,279], where a daily intake of 0.5 mg of MPs causes their deposition in the liver and oxidative stress, thereby resulting in inflammation or disruption of metabolic functions in the liver [280,281,282,283,284]. Research on pluripotent stem cells (PSCs) has revealed that MPs can play a role in the emergence of liver steatosis and fibrosis, along with harmful impacts, as mentioned above, on liver wellbeing [285,286,287]. Regrettably, less is known about the effects of MPs on the human liver, and these should be investigated to determine the toxicity and mechanisms of their impacts on the liver [154].

#### 3.4.8. Skin

Regarding skin absorption, MPs enter the human body through the skin less quickly than other exposure modes. Based on the information in Figure 5B, the MPs being studied can penetrate the skin’s protective barrier and cause cellular interactions. Despite one study showing that MPs larger than 100 μm do not enter the body through skin absorption [180], other evidence suggests that nano-plastics (4–45 nm in size) may still enter the surface layer of the skin [180,288] and cause oxidative stress in human epithelial cells, in addition to inducing inflammation [264,289]. Notably, before 2019, most commercially available personal care and cosmetics products (PCCPs) contained plastic microbeads [290]. According to a study, the average American used 2.4 mg of MPs per person per day in 2011 [291]. In 2016, 69% of PCCPs in Macau contained microbeads, where PE was the most common ingredient [290]. Moreover, in 2017, Lei et al. [292] reported the microbead content of market PCCPs in Beijing and found that face wash was 7.1% MPs (MP size of 313 µm and content of 25.04 pieces per g), body wash was 2.2% MPs (MP size of 422 µm and less than 18 pieces per g), and toothpaste was not found to contain MPs.

#### 3.4.9. Cancer

MPs have the property of serving as carriers, as highly carcinogenic PAHs are easily adsorbed onto MPs. According to the research conducted by Mastrangelo et al. [293], exposure to a threshold of 0.2 mg/m^3^ of PAHs is considered unsafe, while exposure has been found to increase the risk of lung cancer by 1.2–1.4-folds and bladder cancer by 2.2-folds over 40 years. It was verified by Sharma et al. [294] that the concentration of PAHs in the leachate of MPs was about 46–236 µg g^−1^, thus having the potential risk of inducing cancers (lung, breast, and skin). The available evidence does not establish a conclusive connection between MPs and cancer formation [156]. It will be necessary to conduct extensive research to comprehend these issues in the future thoroughly.

## 4. Current Regulations and Prospects for Plastic Products

### 4.1. Regulations for Plastic Products Used in Various Countries/Regions

Due to the rising awareness of environmental protection, as well as the continuous concern about plastic-related pollution (as mentioned above), relevant plans and corresponding policies have been proposed in the international arena with the expectation of reducing the pressure on resources and the environment and regulating plastic recycling by banning or limiting the production, sale, and use of certain plastic products. Moreover, until the appearance of suitable alternative products, the management of plastic products’ production, circulation, use, recycling, and disposal must be established and improved [295]. A comprehensive report by Jakovcevic et al. [296] showed that levying a fee on disposable plastic bags can significantly impact consumer behavior. At the same time, their study found that external incentives led to a marked increase in the number of shoppers who brought their bags to the store in comparison to supermarkets that did not implement a fee and the surge in the adoption of reusable bags was primarily driven by a desire to protect the environment, but, some stakeholders who opposed the policy contended that it aimed solely to reduce financial costs. According to recent reports, charging a fee for plastic bags in China has decreased their usage by 44%, while the unintended consequence of this policy is that people are now excessively using more free internal plastic bags instead of external ones [147]. Despite the success of the UK’s plastic bag charge, one billion single-use plastic bags are still being purchased each year [297]. The realities mentioned above highlight the disconnect between projected environmental attitudes and real-world environmental behaviors, as well as the need for further measures to address the plastic waste problem and encourage the adoption of sustainable alternatives to protect the environment [147,295,297].

The five continents’ plastic regulations, policies, and countermeasures are listed below. It is encouraging to witness that numerous countries and territories have taken action to combat the problem of plastic waste by enforcing bans and regulations. These initiatives are being implemented worldwide, across all five continents (Table 4), and they contribute to creating opportunities within the global community to improve people’s quality of life. The current state of global plastic regulation policies indicates a notable variation in the degree of activity across the five continents concerning limiting the use of plastics. The Asian region, widely recognized as the most polluted region globally, demonstrates a range of implementation levels for plastic limitation programs among its constituent countries. In the Oceania region, people are highly aware of the environment and are willing to work together to reduce plastic usage; as a result, various measures have been put in place. African countries have more policies for restricting plastic coverage, with almost 60% of nations implementing such procedures.

On the contrary, countries in the Americas have been slower to implement policies without a national timetable for restricting plastics. These countries have taken longer to implement such policies, perhaps due to differences in local economic interests and cultures. It is worth mentioning that Europe is more stringent in policy enforcement. It is worth noting that a study has recommended the establishment of a suitable recycling mechanism for using discarded plastic bags as a sustainable option for electricity production [298]. Therefore, the government requires careful consideration when formulating policies before implementation. Implementing a plastic restriction policy effectively requires coordinated adjustments and cooperation from multiple parties, including the government, enterprises, and consumers, to achieve the plastic reduction goal [295,299].

**Table 4:** Plastic restriction policies and countermeasures on five continents.

Relevant Regulation/Policies and Description		Region	Countries and Regions with the Implementation Year	References Sources
Limiting the use of disposable plastic products	An environmental protection measure for minimizing the impact of plastic waste on the environment by enacting corresponding regulations and laws.	Asia: Several delivery platforms and restaurants have limited or complete prohibitions on providing disposable utensils and straws.	China (2008) Nepal (2011) Indonesia (2016) Thailand (2018) Philippines (2018) Taiwan (2019) Pakistan (2019) Japan (2021) India (2022)	[300,301,302,303,304,305,306,307,308]
		Europe: Serving plastic knives, forks, bowls, plates, and cups at restaurants is prohibited. Denmark was the first country to introduce an upstream tax on imports or at the manufacturing level (plastic bag fee).	Denmark (1994) Portugal (2014) France (2016) Turkey (2019) Ireland (2021) Germany (2021) Italy (2021) Netherlands (2021)	[306,309,310,311,312]
		America: Several cities have imposed bans or restrictions, such as New York’s prohibition on plastic foam containers.	Chile (2018) Peru (2019) Panama (2019) United States (2020) Canada (2021)	[294,306,313]
		Africa: Some countries have implemented prohibitions or restrictions on plastic products, such as Kenya’s prohibition on the use of plastic bags.	Rwanda (2008) Madagascar (2015) Kenya (2017) Tanzania (2019) Senegal (2020) Mauritius (2021)	[301,306,314,315]
		Oceania: Step-by-step approaches have been taken, such as New Zealand’s project to phase out cotton labels and straws.	Niue Island (2005) South Australia (2009) Palau (2017) New Zealand (2019) Tuvalu (2019)	[306,316]
Plastic Bag Charges	This policy will reduce the use of plastic bags, encourage people to reuse bags or other alternatives, shift to an eco-friendlier way of shopping, and raise awareness of environmental protection and sustainable development.	Asia: A fee will be charged to customers who request plastic bags and is enforced by the merchant.	Japan (1991) Taiwan (2002) Hong Kong (2009) Indonesia (2016) Turkey (2019)	[122,304,306,317,318,319]
		Europe: A fee will be charged to customers who request plastic bags, and is enforced by the merchant. Ireland was the first country to introduce a plastic bag tax of EUR 0.22.	Ireland (2002) Italy (2011) United Kingdom (2015) Germany (2016) France (2016)	[306,320,321]
		America: Merchants charge a fee to customers who request plastic bags (prices vary by region). Mexico has implemented a prohibition on the provision of plastic bags by merchants.	Ecuador (2016) Colombia (2017) Costa Rica (2018) Peru (2019) Argentina (2019) Mexico (2020)	[307,321,322,323]
		Africa: A fee will be charged to customers who request plastic bags, enforced by the merchant. Kenya is the first in Africa to prohibit all plastic bags.	South Africa (2003) Madagascar (2015) Mozambique (2016) Kenya (2017) Tunisia (2017)	[301,307,321,324,325,326]
		Oceania: A fee will be charged to customers who request plastic bags and is enforced by the merchant. Samoa has prohibited plastic bags with a thickness of less than 50 µm	Australia (2018) New Zealand (2019) Cook Islands (2019) Vanuatu (2020) Perth (2021)	[306,307,321]
Prohibited products containing plastic microbeads	Prohibition of plastic beads in care and cleaning products because MPs cannot be fully filtered by wastewater stations, leading to serious contamination and hazards while encouraging the development of sustainable alternatives.	Asia: Several countries have made relevant policies for enforcement. Taiwan has implemented the “Law on Prohibiting Microbeads.”	South Korea (2018) Taiwan (2018) India (2018) China (2020) Japan (2022)	[122,303,306,318,327]
		Europe: Policy on reducing the use of plastic beads has been fully implemented	Germany (2018) France (2018) United Kingdom (2018) Sweden (2018) Ireland (2018)	[306,327,328]
		America: Microbeads are prohibited in laundry and personal care products.	United States (2015) Canada (2016) Mexico (2018) Costa Rica (2019) Peru (2020)	[307,327]
		Africa: Several countries have made relevant policies for enforcement. South Africa has implemented the “Wash, Detergent, Cosmetics, and Perfume Act”, which prohibits the use of microbeads.	Kenya (2015) Morocco (2016) South Africa (2018) Egypt (2019) Algeria (2019)	[121,301]
		Oceania: Several countries have made relevant policies for enforcement. New Zealand implemented the “Microbeads Prohibition Act”, which prohibits microbeads in beauty and care products.	NewZealand (2018) Australia (2018) Cook Islands (2018) Samoa (2018) Papua New Guinea (2019)	[306,329]

### 4.2. Reducing Plastics Builds on the Linkage of Environmental, Social, and Governance (ESG) with the Sustainable Development Goals (SDGs) 

Plastic reduction is a closely linked approach to ESG, which is an integrated framework for assessing environmental, social, and governance performance in terms of sustainability, and the following are some of the critical interplaying elements:

The purpose of reducing plastic use is to minimize the production of plastics and MPs while minimizing energy consumption (gas and petroleum) during manufacturing and recycling, thereby reducing CO_2_ discharge and improving the greenhouse effect and other negative impacts [330]. Moreover, reducing MPs improves biological and environmental hazards and protects biodiversity. Celluloid, the predecessor Specimen Banks, has supported the achievement of the goals of the European Green Deal, which was precisely to monitor environmental chemicals to avoid changes affecting biodiversity [331]. Current plastic reduction policies will contribute to the environmental protection targets in ESG. 

The management and treatment of plastic waste come with significant financial and energy expenses. Therefore, these are crucial responsibilities for both the economy and society. Consequently, reducing plastic consumption can mitigate the risks, alleviate their burden on society, enlighten people’s awareness and concern about MPs, and increase social participation. Interest in exploring the development, production, and recycling of alternatives to plastics could also provide relevant employment opportunities, and these are linked to the social objectives of ESG. Moreover, implementing plastic reduction requires corresponding and effective regulations for governance.

In contrast, the linkage between social governance and policymaking is based on information transparency and statistical reporting, which facilitate implementing, monitoring, and evaluating plastic reduction and tracking progress toward achieving the target [330]. Overall, there is a need to maintain a cooperative relationship between the government, non-government organizations, businesses, and other related stakeholders to build trust, cooperation, and cross-boundary partnerships and achieve the key to sharing resources to achieve the plastic reduction milestone [332]. Simultaneously, sustainable development and establishing an effective economy, collectively promoted by these cooperations, will foster a sustainable future. Reducing plastic usage is crucial for achieving the Sustainable Development Goals (SDGs) [333,334]. These goals involve protecting land and sea ecosystems, effectively managing climate and water resources, adopting sustainable consumption and production practices, and promoting sustainable urban development. Recycling and reusing plastic can significantly aid in achieving the SDGs. With ongoing efforts, there will be opportunities to achieve these global goals by 2030.

## 5. Conclusions

By categorizing and organizing evidence, this review demonstrated that it seems inevitable that MPs will continue to increase in the coming years. Still, it is possible to initiate reductions in sources, reduce the reliance on disposable plastic products, and manage waste plastics well, with particular reference to the fact that suitable solutions for the treatment and recycling of plastics have yet to be found. In addition, pyrolyzed MPs enter the human body through the ingestion of contaminated food or drinking water, as well as through respiratory inhalation and dermal contact with the source of contamination, with currently known quantities ranging from 203 to 332 pieces of MPs per person per day. However, there is still no evidence showing that MPs directly harm the human body, but many relevant studies have reported a high potential risk of health hazards from MPs. Moreover, the gastrointestinal tract is especially vulnerable to these risks. In this case, the gastrointestinal tract represents the most direct risk, with a weekly intake of 0.1–5 g of MPs and a maximum daily dose of 9 mg/kg of body weight; in addition, MPs migrate to other organs of the body through the metabolic mechanisms of the gastrointestinal tract.

In summary, MPs are generated through the breakdown of plastic debris from various sources and are subsequently released into the environment via diverse channels. This review aims to offer guidance on the proper handling and disposal of MPs, as well as strategies for limiting the use of plastic products. All nations have been encouraged to adopt policies to reduce the use of plastic products and policies for recycling management to monitor the migration and distribution of MPs, actively control the sources of the MPs, effectively reduce the release of plastic particles into the environment and move towards the common goal of global sustainable development. Simultaneously, these solutions and future research should incorporate an interdisciplinary approach and consider cultural differences. Hence, we must protect our environment and its inhabitants by acting now to prevent harm and eliminate harmful plastic products and MPs.

## Figures and Tables

**Table 1:** History of plastic development.

Chronicle Year	Representative Event		References
15th century	Start using natural rubber	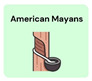	[4]
1839	Co-heating of rubber and sulfur into elastic and plastic polymers	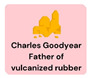	[5]
1869	Created the first synthetic plastic “celluloid.”	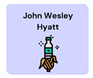	[6,7,8]
1909	Invented the first artificial plastic- phenolic resin. Commonly known as bakelite	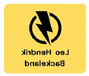	[9,10]
1920	The term polymer and polymer officially appeared	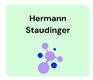	[11]
1926	The earliest synthetic plastic Polyvinyl Chloride	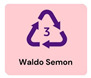	[12,13]
1930	Extracted polystyrene	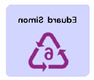	[14]
1933	Synthesized polyethylene	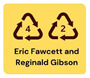	[9,15,16,17]
1954	The advent of polypropylene	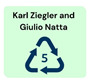	[18,19]
1967	The Birth of PET Bottle—Polyethylene Terephthalate	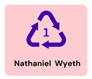	[20,21,22]
2004	Coined the word microplastics	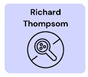	[23]
2016	Microplastic pollution is officially listed as the second-largest issue in environmental and ecological science.	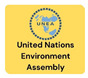	[24,25,26]
2023	Taiwan banned the use of polyvinyl chloride in food packaging starting in July.	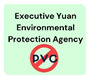	[27]

**Table 2:** Differentiating plastic debris in the ocean according to particle size and the difference between primary and secondary microplastics.

Plastic Classification Level	Subcategory	Size	Representative Item	Symbolic Picture
Macroplastic	-	25 mm	Plastic bottle	-
Mesoplastic	-	2–25 mm	Plastic debris	-
Microplastic	Primary Microplastics	1 µm–5 mm	Microbeads	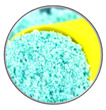
			Colloidal particles	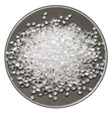
	Secondary Microplastics		Net	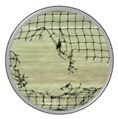
			Tires	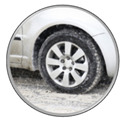
Nanoplastic	Invisible to the naked eye	<1 µm	-	-

**Table 3:** Comparison of degradation methods.

Category	Degradation Method	Advantages	Disadvantages	References
Physical degradation	Thermal degradation Mechanical degradation	Ease of operation	Limited scope of application Toxic gas release Not easily degraded completely	[61,63,64,80]
		Takes less time	Limited scope of application Toxic gas release Not easily degraded completely	
		Can recover energy	Limited scope of application Toxic gas release Not easily degraded completely	
Chemical degradation	Photodegradation	More environmentally friendly	Incomplete degradation Time-consuming Affected by the environment	[64,86,89]
		High versatility	Incomplete degradation Time-consuming Affected by the environment	
			Incomplete degradation Time-consuming Affected by the environment	
Biodegradation	Microbial decomposition Metabolic Mechanisms in Organisms	More environmentally friendly	Potentially harmful to organisms in case of incomplete degradation	[94,97,98,105,106]
		Less extra pollution	Affected by the environment	
		It can be used in a targeted manner	Influenced by microbiota	

## Data Availability

Not applicable.
